# LncRNA MALAT1 Regulating Lung Carcinoma Progression via the miR-491-5p/UBE2C Axis

**DOI:** 10.3389/pore.2021.610159

**Published:** 2021-03-29

**Authors:** Juanjuan Dai, Ning Zhou, Rui Wu, Jing Du, Shuang Miao, Kaikai Gong, Lijuan Yang, Weiwei Chen, Xuelin Li, Chen Li, Yan Wu

**Affiliations:** ^1^ Cancer Research Institute, Binzhou Medical University Hospital, Binzhou, China; ^2^ Department of Otolaryngology Head and Neck Surgery, Binzhou Medical University Hospital, Binzhou, China; ^3^ Institute for Metabolic and Neuropsychiatric Disorders, Binzhou Medical University Hospital, Binzhou, China

**Keywords:** MALAT1, miR-491-5p, UBE2C, lung carcinoma, proliferatiom

## Abstract

Long noncoding RNAs (lncRNAs) play a critical role in the development of lung carcinoma. The mechanism of MALAT1 in lung carcinoma development is not understood very well. This study aimed to investigate the role of MALAT1 in lung carcinoma progression and the mechanism underlying the role of miR-491-5p in the MALAT1 mediated regulation of UBE2C expression. The results indicated that the expression of MALAT1 was often augmented in lung carcinoma cells. Suppression of MALAT1 blocked the proliferation, invasion and migration ability of cancer cells and inhibited the expression of UBE2C. UBE2C restoration attenuated the MALAT1 knockdown-induced anti-cancer effects. Moreover, UBE2C and MALAT1 were indicated as targets of miR-491-5p and inhibition of miR-491-5p restored the MALAT1 knockdown-induced inhibition of the progression of lung carcinoma. Furthermore, MALAT1 sponged miR-491-5p to upregulate UBE2C expression, causing it to act as a competing endogenous RNA. Collectively, MALAT1 downregulation suppressed lung carcinoma progression by regulating the miR-491-5p/UBE2C axis. These results indicate that MALAT1 could be a molecular target for lung carcinoma treatment and prognosis.

## Introduction

Lung carcinoma is among the leading causes of cancer-related deaths in China and worldwide [1, 2]. Despite the progression in diagnosis, chemotherapy and immunotherapies, the overall survival rate of lung carcinoma is still relatively low [3, 4]. Further investigation of the molecular mechanisms involved in the disease, in particular, those associated with its pathology, is imperative to allow the accumulation of knowledge regarding this condition and to focus on personalized therapy.

Long non-coding RNAs (lncRNAs) have little or no protein-coding capacity and their length is >200 nucleotides [5]. Recent studies have indicated that lncRNAs may affect many cellular physiological processes, including the cell cycle, apoptosis, migration, senescence and chemoresistance [6–9]. With a length of >8,000 nucleotides, MALAT1 (metastasis-associated lung adenocarcinoma transcript 1), also known as NEAT2 (nuclear enriched abundant transcript 2), is one of the first tumor metastasis-associated and highly conserved lncRNAs to be discovered. Previously, a large amount of evidence has shown that MALAT1 plays an important role in the tumorigenesis of many types of tumors, including lung carcinoma, breast cancer and pancreatic cancer [10–12]. Abundant research has indicated that MALAT1 acts as a “sponge,” regulating microRNA activity, which in turn leads to the regulation of the expression of genes affecting cell proliferation, apoptosis, migration and invasion [13]. For example, MALAT1 downregulates miR-145 to modulate endothelial-to-mesenchymal transition induced by TGF-β1 in neointimal hyperplasia [14]. For sponging of miR-23b-3p, MALAT1 acts as a competing endogenous RNA (ceRNA), reducing the degradation of ATG12 induced by miR-23b-3p, causing autophagy and drug-resistance in gastric cancer cells [15]. UBE2C (ubiquitin-conjugating enzyme 2C), also known as UBCH10, is upregulated during carcinogenesis and development of various human cancers including lung carcinoma [16, 17]. Furthermore, several studies have demonstrated that UBE2C acts as an underlying oncogene in lung carcinoma, regulating the cell cycle, proliferation, migration, autophagy and chemoresistance of lung carcinoma cells [18–20].

In this study, we provide that MALAT1 could upregulate the expression of UBE2C. We also carried out an analysis of the putative binding sites between MALAT1 or UBE2C and miR-491-5p using bioinformatic methods. Hence, we hypothesized that MALAT1 might act as a ceRNA for sponging miR-491-5p and controlling UBE2C expression to regulate the progression in a substantial number lung carcinoma. In this study, we detected MALAT1 expression level in lung carcinoma cell lines and the role of MALAT1 in cell proliferation, colony formation, migration and invasion were examined. Moreover, we investigated the ceRNA-regulating mechanism of the MALAT1/miR-491-5p/UBE2C system.

## Materials and Methods

### Cell Culture and Transfection

All human lung cancer cell lines (H1299, H520, H385, calu6, H460, SW900 and 95D) and HBEC (Human Bronchial Epithelial Cells) were purchased from ATCC (American Type Culture Collection) and cultured in RPMI medium containing 10% FBS (Hyclone, United States) and 1% PS (penicillin and streptomycin).

The pcDNA3.1 and pcDNA3.1-UBE2C vector was stored in our lab and the stable UBE2C overexpressing cell lines were selected using aminoglycoside antibiotic G418 (geneticin) reagent. The constructs of wild-type (WT) or miR-491-5p binding site mutant type (MUT) 3′-UTR on MALAT1 or on UBE2C were constructed using the pMIR-reporter luciferase vector (Vigene Rockville, NZ, United States). Short hairpin RNA targeting MALAT1 (shMALAT1: 5′-AGA​UAU​UUA​AGA​ACT​CCA​CAG​C-3′), a negative control (NC: 5′-ACG​UGA​CAC​GUU​CGG​AGA​AC-3′), miR-491-5p mimics (5′- CCU​CAU​GGA​AGG​GUU​CCC​CAC​U-3′) or miR-491-5p inhibitor (5′-TGU​GGG​AAC​CCU​UCC​AUG​AGG-3′) and the miRNA-NC (5′- GUC​UCC​ACG​CGC​AGU​ACA​UUU-3′) were purchased from GenePharma (Shanghai, China). Cells were transfected using Lipofectamine 3,000 reagents (Invitrogen) following the manufacturer’s protocol. The cells were harvested at different time points for analysis.

### Cell Proliferation Assay

Cells (2 × 10^3^) transfected with the relevant plasmid were seeded into 96-well plates for three duplicates and cultured in an incubator for 24 h. According to the manufacturer’s specifications, 10 μL of cell counting kit-8 (CCK-8, Dojindo, CK04) reagent was added, followed by incubation for 3 h. A spectrophotometer was used to measure the optical density of the samples at 450 nm and 630 nm. Three times independent experiments were repeated.

### Western Blotting

Tissues and cells were harvested, lyzed in lysis buffer and incubated on ice for 30 min. The total protein concentration was measured by BCA Protein Assay Kit (Sangon, China) before separation by electrophoresis. 30 μg protein/sample was loaded into the SDS-PAGE gel. After transferring, the membranes were incubated with Tris buffer containing 5% non-fat dry milk and 0.1% Tween-20 at 25°C for 1 h. The membranes were then incubated with the following primary antibodies: anti-UBE2C (ab12290, Abcam, Cambridge, United Kingdom), anti-tubulin (ab6046, Abcam, Cambridge, United Kingdom) or anti-β-actin (YT0099, Immunoway, TX, United States), then incubated with HRP-conjugated goat anti-rabbit secondary antibodies (ab6721, Abcam, Cambridge, United Kingdom) at room temperature for 1 h. The immunoreactive protein bands were developed and analyzed using the ECL substrate kit (Millipore, United States).

### RT-PCR

RNA was extracted with Eastep™ total RNA extraction kit (Promega, United States) and reverse transcribed into cDNA with RevertAid First Strand cDNA Synthesis Kit (ThermoFisher, United States) following the manufacturer’s instructions. PCR was performed with 2×EasyTaq^®^ PCR SuperMix (TransGen Biotech, China). Amplification was performed as follows: a denaturation step at 94°C for 5 min, followed by 30 cycles of amplification at 94°C for 30 s, 55°C for 30 s, 72°C for 30 s. Three parallel reactions were performed on GeneAmp^®^ PCR System 9,700 (Applied Biosystems, United States). GAPDH was used as a reference gene. Primers used in the RT-PCR were as follows: UBE2C FP: 5′- GGA​TTT​CTG​CCT​TCC​CTG​AA-3′, UBE2C RP: 5′-GAT​AGC​AGG​GCG​TGA​GGA​AC-3′, MALAT1 FP: 5′-GCG​ACG​AGT​TGT​GCT​GCT​ATC​T-3′, MALAT1 RP: 5′-ACA​CTG​CTC​TGG​GTC​TGC​TTT​T-3′, GAPDH FP: 5′-CTC​CTC​CTG​TTC​GAC​AGT​CAG​C-3′, GAPDH RP: 5′-CCC​AAT​ACG​ACC​AAA​TCC​GTT-3′.

### Transwell Assay

As matrigel simulates the extracellular matrix, a 24-well Transwell chamber (Costar 3,422, Corning, NY, United States) with or without a matrigel-coated membrane (BD Bioscience, CA, United States) was used to perform the cell invasion and cell migration assays, respectively. Two parallel chambers were performed for each group. Following transfection, 2 × 10^3^ cells (H1299 and H520) were suspended with serum-free RPMI medium before been seeded into the upper chambers. The chambers were maintained in wells containing medium and 20% FBS. After incubation for 48 h, cotton swabs were used to remove the cells that were still in the membrane of the chamber. After fixing and staining with 4% paraformaldehyde and crystal violet, the membrane was photographed using an Olympus light microscope (Olympus, Japan). The cells in the 4 non-repeating field were randomly selected and counted.

### Wound-Healing Assay

The 5 × 10^5^ cells/well were seeded in 6-well plates and cultured for 24 h. The cell monolayers at the bottom of the plate were scratched with sterile 200 μL pipette tips before washing twice with PBS buffer. The cells were cultured in RPMI medium without FBS. A light microscope (Olympus, Japan) was used to take photographs at 0 h and 48 h after the establishment of the wound (scratching). Experiments were performed in triplicates.

### Colony Formation Assay

Following transfection, the cells were seeded into 6-well plates (at density of 700 cells/well) and cultured for another two weeks. The experiments were performed as triplicate. Cell colonies were fixed with 4% paraformaldehyde for 20 min and stained with crystal violet (Sangon) for 15 min prior to optical imaging.

### Cell Cycle Analysis

After 48 h of transfection, cells were collected and washed with cold PBS. Cells were resuspended in 300 μL of a propidium iodide (PI) mixture (containing 200 μg/ml PI, 100 μg/ml RNase and 0.2%Triton X-100) and incubated at 4°C for 30 min. Fluorescent signals were measured at the FL-2 channel using CFlow Plus package from Accuri C6. The data obtained analyzed using ModFit software. Experiments were performed in triplicates.

### Analysis of Publicly Available Datasets

The online web server GEPIA (Gene Expression Profiling Interactive Analysis, http://gepia.cancer-pku.cn/index.html) was used to analyze the UBE2C expression level in human lung carcinoma.

### Dual-Luciferase Reporter Assay

The MALAT1 or UBE2C 3′-UTRs or the mutant of miR-491-5p binding sites were cloned into the pMIR-reporter luciferase vector (Vigene, Rockville, NZ, United States) using *Hind* III and *Sac* I restriction enzyme sites to generate the MALAT1 3′-UTR wild-type (WT), MALAT1 3′-UTR MUT type, UBE2C 3′-UTR wild-type (WT) and UBE2C 3′-UTR MUT type plasmids. The recombinant plasmids and miR-491-5p mimics were co-transfected into 3T3 cells using Lipofectamine 3,000. Luciferase activity was measured using a dual-luciferase detection kit (Promega, Madison, WI, United States), according to the manufacturer’s protocol.

### Human Lung Tumor Tissue Collection

All the tumor and normal lung tissues of human were gathered from Binzhou Medical University Hospital which were consented and approved from the patients and Institute Research Ethics Committee (2019-G019-01).

### Data Analysis

Organizing by Graphpad Prism 7.0, the results were presented as the mean ± SD. Two-tail Student’s *t*-test and ANOVA with Tukey post-hoc test were used to compare two groups and multiple groups, respectively. *p* < 0.05 represented the statistical significance.

## Results

### Upregulation of MALAT1 in Lung Carcinoma Tissues and Cell Lines

**FIGURE 1:**
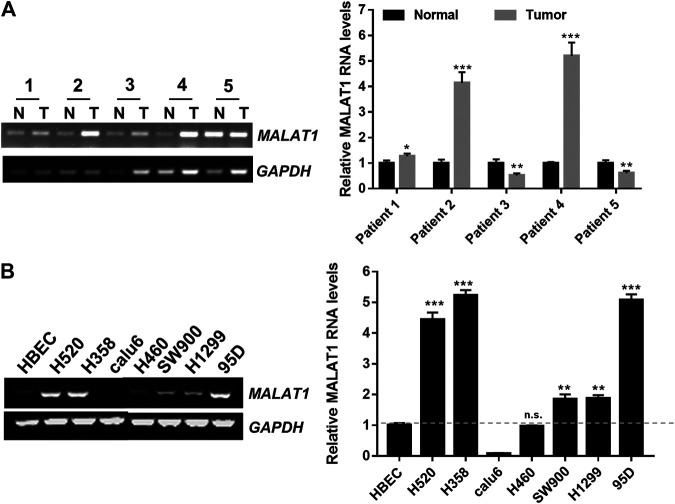
Up-regulation of MALAT1 in lung carcinoma **(A)** RT-PCR indicated that MALAT1 was overexpressed in human lung carcinoma tissues, compared to the adjacent lung tissues (N: normal tissues, T: tumor tissues). **(B)** Gel-based RT-PCR assay demonstrating that the expression of MALAT1 was highly increased in lung cancer cell lines, compared to that in HBECs (Human Bronchial Epithelial Cells). The dashed line represented the reference value (^*^ *p* < 0.05, ^***^ *p* < 0.001).

An RT-PCR assay was performed to examine the MALAT1 expression levels in cancer tissues obtained from lung cancer patients, the MALAT1 RNA levels were higher in tumor tissues than in adjacent tissues (Figure 1A). Endogenous MALAT1 RNA levels were also markedly higher in H520, H358, SW900, H1299 and 95D cells than in human bronchial epithelial cells (HBECs) (Figure 1B).

### Downregulation of MALAT1 Suppresses the Proliferation, Migration and Invasion Ability in Lung Carcinoma Cells

**FIGURE 2:**
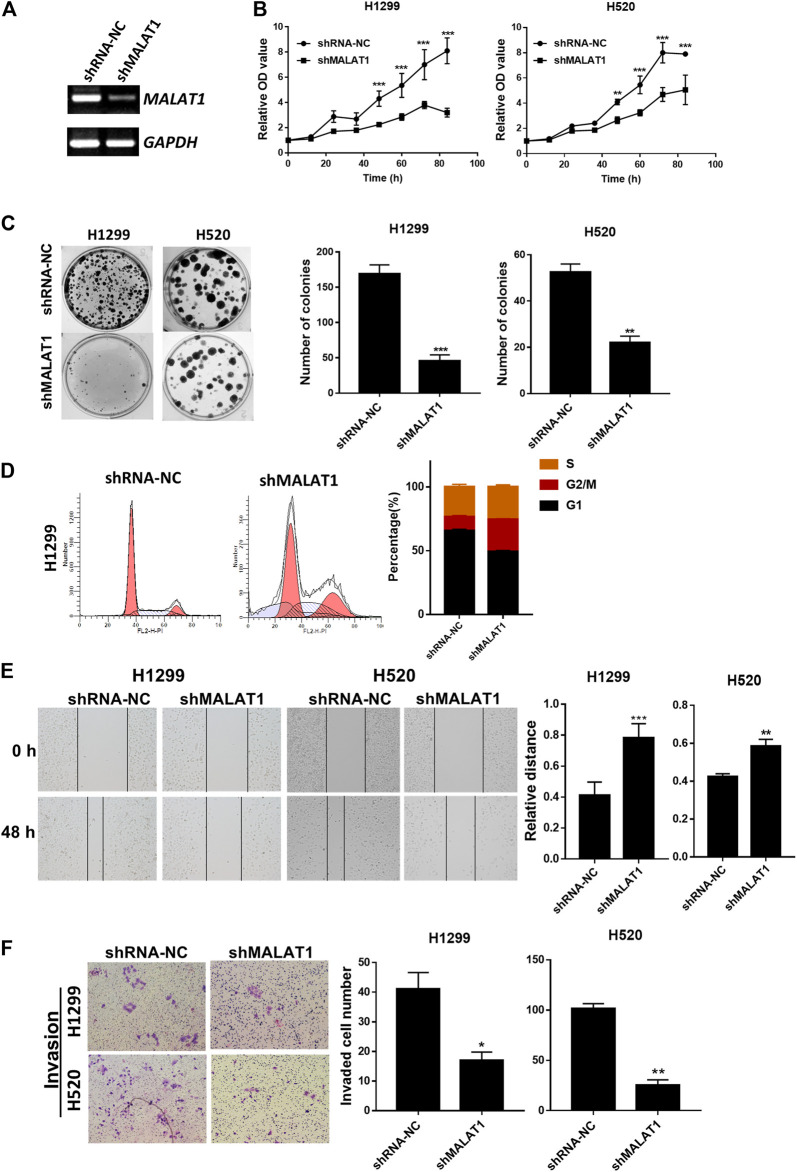
Knockdown of MALAT1 suppressed the progression of lung cancer cells. **(A)** Gel-based RT-PCR showed that the knockdown of MALAT1 decreased the RNA level of MALAT1 in H1299 cells. **(B)** CCK-8 assay demonstrating that blocking the expression of MALAT1 markedly arrested the proliferation of H1299 and H520 cells. **(C)** Knockdown of MALAT1 repressed colony formation of H1299 and H520 cells. **(D)** Cell cycle analysis was performed to analyze the distribution of H1299 cells in various phases of the cell cycle after MALAT1 knockdown. **(E)** The wound-healing assay demonstrated that the knockdown of MALAT1 markedly retarded scar healing at 48 h in H1299 and H520 cells. **(F)** Transwell assay performed to evaluate the invasion ability of H1299 and H520 cells after shMALAT1 treatment (^*^ *p* < 0.05, ^**^ *p* < 0.001, ^***^ *p* < 0.0001).

ShRNA specifically designed to silence MALAT1 was used to investigate whether the expression level of MALAT1 was associated with the proliferation, metastasis and invasion abilities of lung cancer cells. ShMALAT1-1 and shMALAT1-2 were transfected into H1299 cells. As shown in Figure 2A, compared to the shRNA-NC (negative control shRNA) group, MALAT1 expression was significantly decreased in the shMALAT1-2 group. So, the shMALAT1-2 (simply named as shMALAT1) would be applied in this study. CCK-8 and colony formation assays showed that downregulation of MALAT1 notably inhibited the proliferation and colony formation abilities of H1299 and H520 cells (Figures 2B,C). Flow cytometry analysis was used to determine the mechanisms underlying the MALAT1-mediated inhibition of the proliferation and clonal expansion abilities of the cells. The results indicated that knockdown of MALAT1 significantly arrested the cell cycle of H1299 cells in the G2/M phase and increased the cell number of sub-G1 (Figure 2D). The percentage of sub-G1 cells is an indication of the apoptotic cells which arise as a result of MALAT1 knockdown. As indicated in Figures 2E,F, suppression of MALAT1 expression significantly decreased the migration and invasion ability of H1299 and H520 cells.

### UBE2C is Involved in MALAT1-Induced Proliferation, Migration and Invasion of Lung Carcinoma Cells

**FIGURE 3:**
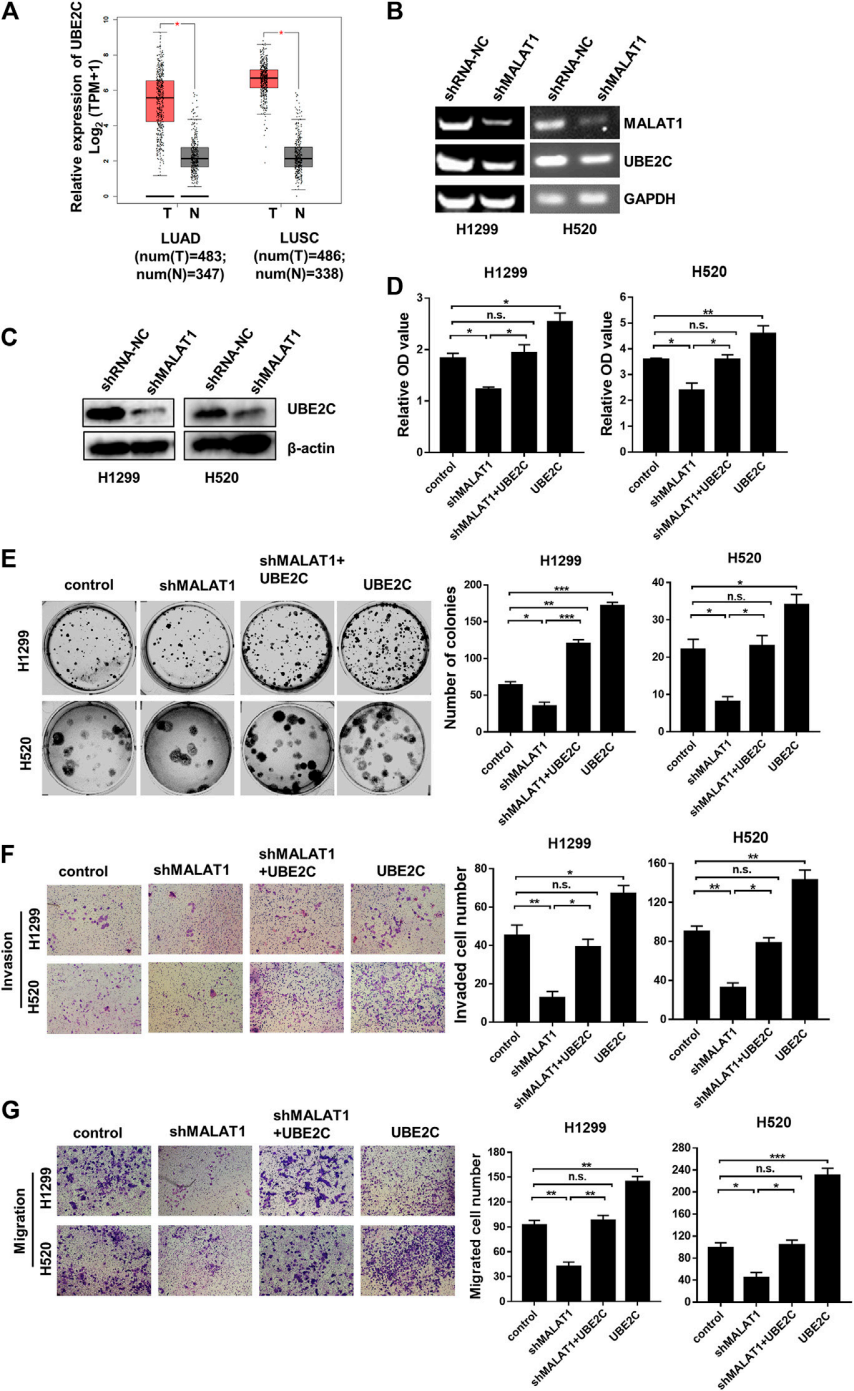
UBE2C overexpression reverses the MALAT1 knockdown-induced inhibition on lung cancer cells. **(A)** The expression levels of UBE2C was increased in both LUAD (lung adenocarcinoma) and LUSC (Lung squamous cell carcinoma) tissues which were analyzed TCGA database using online web server GEPIA (N: normal tissues, T: tumor tissues, TPM: Transcripts Per Million). **(B)** The UBE2C mRNA expression was detected using Gel-based RT-PCR following transfection with shMALAT1 or shRNA-NC (negative control RNA) into H1299 and H520 cells. **(C)** The UBE2C protein expression level was detected by the western blotting assay. **(D)** Overexpression of UBE2C restored the MALAT1 knockdown-induced inhibition of cell proliferation which were detected at 72 h using CCK-8 assay. **(E)** The photograph of the colony formation assay of H1299 and H520 cells transfected with shMALAT1 and/or UBE2C plasmid. **(F,G)** Transwell assay with **(F)** or without **(G)** matrigel for the evaluation of the invasion and migration ability of H1299 and H520 cells following their transfection with the control, shMALAT1 and/or UBE2C plasmid. Control group: co-transfected with shRNA-NC and pcDNA3.1; shMALAT1 group: co-transfected with shMALAT1 and pcDNA3.1; UBE2C group: co-transfected with shRNA-NC and pcDNA3.1-UBE2C; shMALAT1+UBE2C group: co-transfected with shMALAT1 and pcDNA3.1-UBE2C (^*^ *p* < 0.05, ^**^ *p* < 0.01, ^***^ *p* < 0.001).

The results in Figure 2D indicated that the expression level of MALAT1 was strongly linked to the cell cycle of lung carcinoma cells. UBE2C, a gene whose translation product is an anaphase promoting complex/cyclosomes (APC/C)-specific E2 ubiquitin conjugating enzyme to play a vital function in cell cycle regulation [21]. In our previous reports [18], the UBE2C mRNA and protein levels were markedly increased in H1299 and H520 cells compared to the HBEC cells. We speculated that UBE2C is involved in lung carcinoma progression, which is regulated by MALAT1. The online web server GEPIA (Gene Expression Profiling Interactive Analysis, http://gepia.cancer-pku.cn/index.html) was used to further investigate the UBE2C expression levels in lung carcinomas. As shown in Figure 3A, UBE2C was significantly upregulated in LUAD (lung adenocarcinoma) and LUSC (Lung squamous cell carcinoma) compared to the normal tissues. MALAT1 knockdown significantly decreased the expression of UBE2C in H1299 and H520 cells (Figures 3B,C). The CCK-8 assay (Figure 3D) demonstrated that UBE2C overexpression promoted the proliferation of H1299 and H520 cells whereas MALAT1 knockdown blocked the growth of these two cell lines. Elevation of the expression of UBE2C partially rescued cellular proliferation inhibited by MALAT1 knockdown in both H1299 and H520 cells. Moreover, UBE2C accumulation partially restored the cell migration (Figure 3E), clonogenicity (Figure 3F) and invasive growth (Figure 3G) inhibited by MALAT1 down-regulation in H1299 and H520 cells. All these results confirmed that MALAT1 was involved in H1299 and H520 cell progression by regulating the expression of UBE2C.

### MALAT1 Competes With UBE2C for miR-491-5p Binding

**FIGURE 4:**
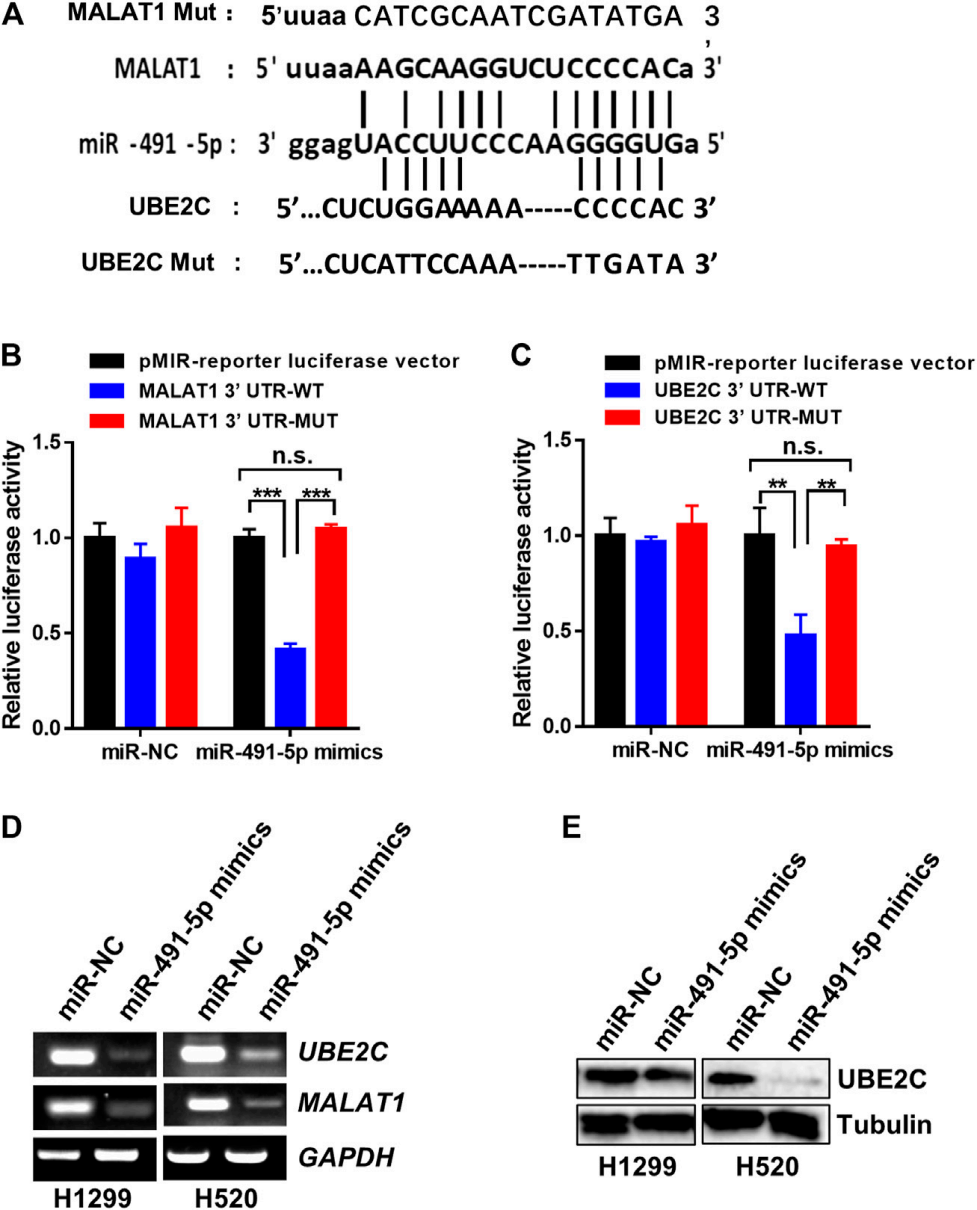
MiR-491-5p directly interact with the RNA of MALAT1 and UBE2C. **(A)** The putative miR-491-5p-binding site and the mutation sequence in the 3′-UTR region of the MALAT1 and UBE2C mRNAs. **(B)** Luciferase reporter assays verified the binding activity between miR-491-5p and the 3′-UTR of MALAT1. **(C)** Luciferase reporter assays analyzed the interaction between miR-491-5p and the 3′-UTR of UBE2C. **(D)** Gel-based RT-PCR assay was used to monitor the inhibitory effect of the miR-491-5p mimics on MALAT1 or UBE2C RNA expression in H1299 and H520 cells. ^*^ *p* < 0.05 vs control, ^**^ *p* < 0.01, ^***^ *p* < 0.001. **(E)** Western blot assay was performed to monitor the inhibitory effect of the miR-491-5p mimics on the UBE2C protein expression in H1299 and H520 cells.

The analysis using the starBase v2.0 software shows that miR-491-5p binds to both MALAT1 and UBE2C in human lung carcinoma cells and the potential sequences responsible for the binding between miR-491-5p and MALAT1 or UBE2C are shown in Figure 4A. To confirm the binding activity between miR-491-5p and MALAT1 or UBE2C, a dual-luciferase reporter assay was performed in 3T3 cells. The dual-luciferase reporter assay indicated that the luciferase activity of cells transfected with the MALAT1 WT, but not the MALAT1 MUT plasmid, decreased significantly following transfection with the miR-491-5p mimics (Figure 4B). Similar results were also observed in the UBE2C 3′-UTR luciferase reporter assay. MiR-491-5p mimics significantly reduced the luciferase activity of the UBE2C WT group, compared to the control group, however this reduction was not observed in the UBE2C MUT group (Figure 4C). To further understand the regulatory relationship between MALAT1, UBE2C and miR-491-5p, RT-PCR and western blotting were performed following the transfection experiment. As shown in Figure 4D, miR-491-5p mimics significantly repressed mRNA expression levels of MALAT1 and UBE2C and downregulated the UBE2C protein levels (Figure 4E).

### Knockdown of MALAT1 Restored the miR-491-5p Inhibitor-Mediated Acceleration of Lung Carcinoma Cell Proliferation, Migration and Invasion

**FIGURE 5:**
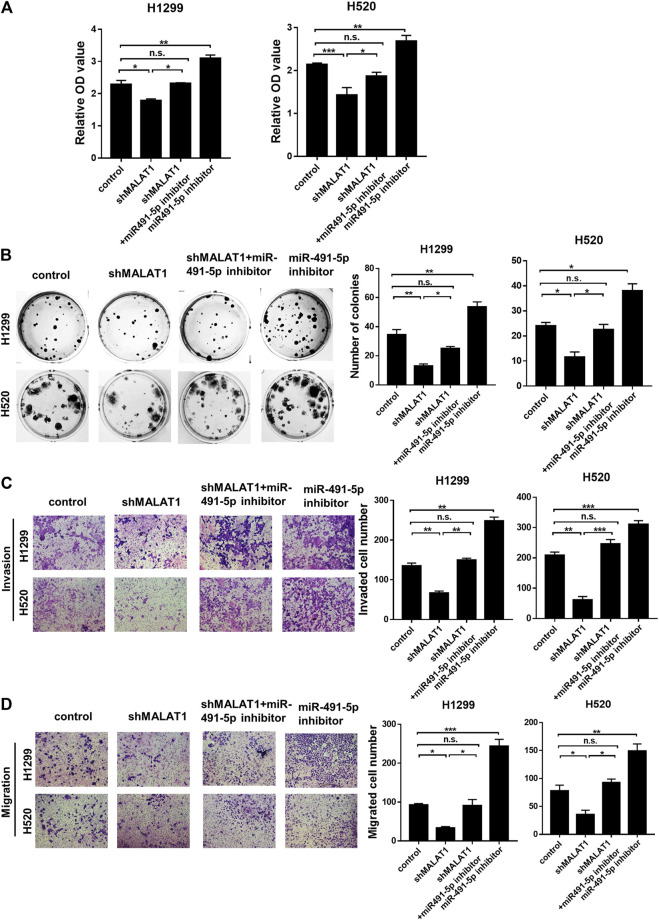
Knockdown of MALAT1 inhibited the progression of lung cancer, which was induced by the miR-491-5p inhibitor. **(A)** CCK-8 assay was performed to evaluate cell proliferation. **(B)** Colony formation assay demonstrating that the miR-491-5p inhibitor markedly increased the MALAT1 knockdown-induced suppression of colony formation ability in H1299 and H520 cells. **(C,D)** Transwell assay performed with **(C)** or without matrigel **(D)** indicated that the miR-491-5p inhibitor promotes the invasion and migration abilities of H1299 and H520 cells via the regulation of MALAT1. ^*^ *p* < 0.05, ^**^ *p* < 0.01, ^***^ *p* < 0.001. Control group: co-transfected with shRNA-NC and negative inhibitor; shMALAT1 group: co-transfected with shMALAT1 and negative inhibitor; miR491-5p inhibitor group: co-transfected with shRNA-NC and miR491-5p inhibitor; shMALAT1+ miR491-5p inhibitor group: co-transfected with shMALAT1 and miR491-5p inhibitor.

**FIGURE 6:**
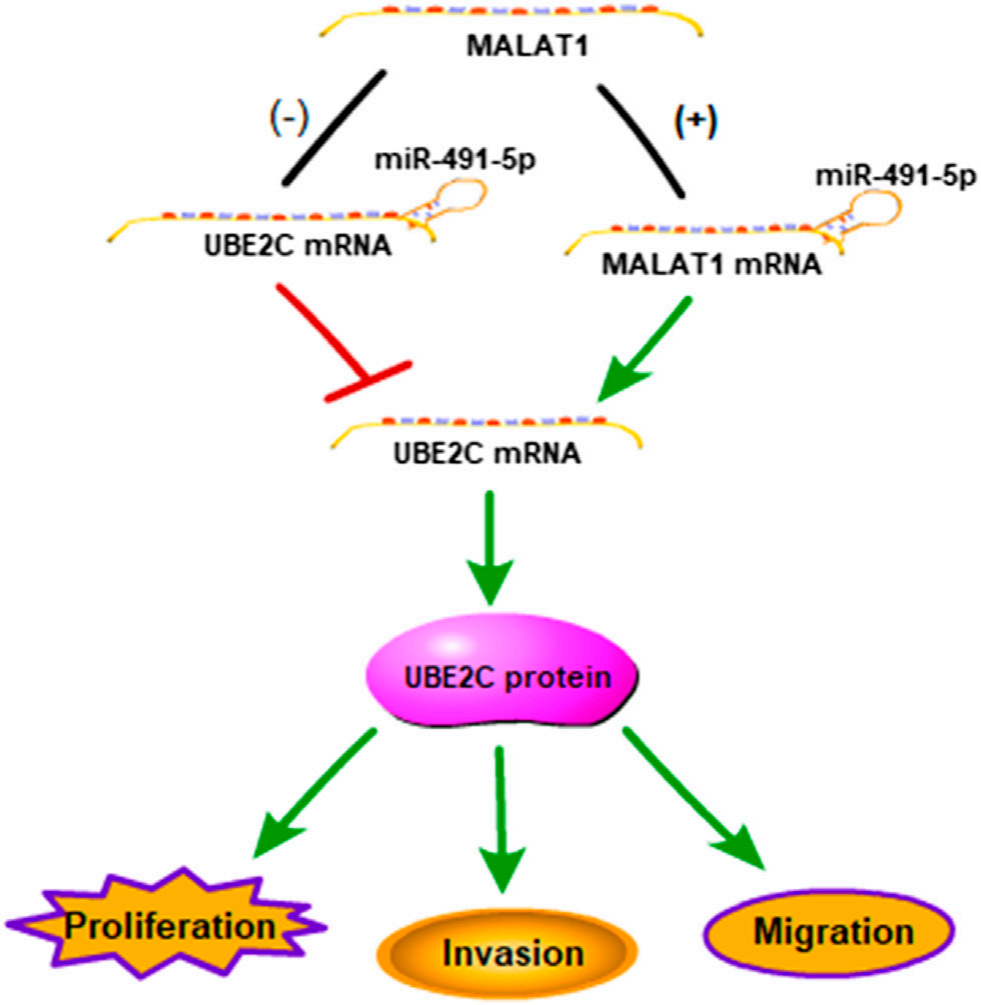
Schematic representation of a model depicting the molecular mechanisms underlying the role of the MALAT1-miR-491-5p-UBE2C system in lung carcinoma.

To determine the mechanism by which miR-491-5p regulates the lung carcinoma progression, H1299 and H520 cells transfected with miR-491-5p inhibitor or co-transfected with shMALAT1 were analyzed for the effects of miR-491-5p on MALAT1-mediated cell proliferation, migration and invasion. The CCK-8 assay showed that the knockdown of miR-491-5p significantly promoted the viability of H1299 and H520 cells, compared to the control group. Down-regulation of MALAT1 markedly suppressed miR-491-5p inhibitor-induced promotion of cell proliferation (Figure 5A). Furthermore, a colony formation assay also demonstrated that the repression of MALAT1 expression significantly reduced the promotion of cell proliferation induced by transfection with the miR-491-5p inhibitor (Figure 5B). Transwell migration and invasion assays demonstrated that the miR-491-5p inhibitor notably promoted the migration and invasion of H1299 and H520 cells, compared to the control group. MALAT1 downregulation significantly attenuated the migration and invasion which were mediated by the miR-491-5p inhibitor in H1299 and H520 cells (Figures 5C,D). These results indicated that the knockdown of MALAT1 attenuated the miR-491-5p-induced promotion of the proliferation, migration and invasion abilities of lung carcinoma cells. Collectively, these findings suggest that MALAT1 stimulates lung carcinoma progression via the miR-491-5p/UBE2C axis (Figure 6).

## Discussion

The long noncoding RNA MALAT1 is overexpressed in numerous cancers and plays a key role in cell proliferation and metastasis via the modulation of the PI3K/AKT [22], NF-kB [23], WNT/β-catenin [24], MAKP/ERK [25] and other molecular signaling pathways. It has been reported that MALAT1 may be a biomarker for overall survival, metastasis-free survival with early-stage NSCLC and for indicating poor clinical prognosis [26]. In this study, we showed that MALAT1 knockdown blocked the proliferation, colony formation, migration and invasion ability of lung cancer cells through the downregulation of expression levels of UBE2C in a substantial number lung cancer cell. However, the potential mechanism is still unknown. The ceRNA-associated mechanism has become one of the crucial regulatory pathways for lncRNAs involved in tumor processes. Therefore, we hypothesized that MALAT1 may act as a ceRNA to modulate UBE2C expression in regulating lung carcinoma progression. Herein, for the first time, we indicated that MALAT1 could sponge miR-491-5p to regulate UBE2C expression.

As an important member of the ubiquitin-proteasome system, UBE2C (Ubiquitin-conjugating enzyme 2C) acts as a ubiquitin-conjugating enzyme to promote cell cycle progression. Our previous studies have indicated that UBE2C participates in various biological processes, such as proliferation, autophagy [18] and chemoresistance [19, 20] and is significantly overexpressed in many types of solid tumors. Aberrant expression of UBE2C leads to the degradation of pVHL (von Hippel-Lindau tumor suppressor), which increases HIF-1α levels, resulting in endothelial inflammation and epithelial-mesenchymal transition in calcific aortic valve disease [27]. The results of this study showed that MALAT1 knockdown decreased UBE2C expression at mRNA and protein levels and UBE2C contributed to the proliferation and colony formation abilities, which were regulated through MALAT1, of lung cancer cells.

Many studies have showed that miR-491-5p acts as a tumor suppressor in several types of cancer, including gastric cancer [28], oral squamous cell carcinoma [29], prostate cancer [30], lung carcinoma [31] and breast cancer [32]. However, the mechanism underlying the role of miR-491-5p in lung cancer is not yet fully understood. The results of this study indicated that the overexpression of miR-491-5p could suppress the expression of UBE2C and MALAT1. Additionally, miR-491-5p was required for MALAT1-UBE2C axis-induced tumorigenesis, cell proliferation and metastasis. Taken together, we demonstrated that the lncRNA MALAT1 is involved in lung carcinoma development by regulating the expression of miR-491-5p and UBE2C.

In conclusion, knockdown of MALAT1 which is upregulated in lung cancer, inhibited the proliferation, colony formation, migration and invasion abilities of lung cancer by sponging miR-491-5p to downregulate UBE2C expression. This study explored the ceRNA regulatory mechanism of the MALAT1/miR-491-5p/UBE2C system, providing a potential therapeutic target for lung carcinoma.

## Data Availability

The original contributions presented in the study are included in the article/Supplementary Material, further inquiries can be directed to the corresponding author.
